# Reduced insulin clearance is linked to subclinical atherosclerosis in individuals at risk for type 2 diabetes mellitus

**DOI:** 10.1038/s41598-020-80581-x

**Published:** 2020-12-31

**Authors:** Elko Randrianarisoa, Angela Lehn-Stefan, Anja Hieronimus, Robert Wagner, Jakob Maucher, Kilian Rittig, Bernd Balletshofer, Andreas L. Birkenfeld, Andreas Peter, Norbert Stefan, Hans-Ulrich Häring, Andreas Fritsche, Martin Heni

**Affiliations:** 1 Institute of Diabetes Research and Metabolic Diseases (IDM) of the Helmholtz Center Munich at the University of Tübingen, Otfried-Müller-Str. 10, 72076 Tübingen, Germany; 2 German Center for Diabetes Research (DZD), Tübingen, Germany; 3 Department of Internal Medicine IV, Division of Endocrinology and Diabetology and Nephrology, University Hospital of Tübingen, Tübingen, Germany; 4 Department for Diagnostic Laboratory Medicine, Institute for Clinical Chemistry and Pathobiochemistry, University Hospital of Tübingen, Tübingen, Germany

**Keywords:** Diabetes, Cardiovascular diseases, Metabolic disorders

## Abstract

Hyperglycemia and insulin resistance contribute to vascular damage and are regulated by different pathophysiological processes. The aim of the study was to systematically investigate the relative contributions of multiple fasting state- and oral glucose tolerance test (oGTT)-derived glycemic traits to carotid intima-media thickness (cIMT), a surrogate parameter of subclinical atherosclerosis, in individuals with increased risk for type 2 diabetes mellitus (T2D). 667 volunteers (417 women and 250 men, mean age 44.1 years), who were free of cardiovascular disease (CVD), were included in this cross-sectional study. Glucose tolerance, insulin sensitivity, insulin secretion and insulin clearance were assessed by frequently sampled 75 g oGTT. CIMT was measured by high-resolution ultrasound. Insulin clearance was associated with cIMT in univariate analysis (ß_st_ = − 0.17, p < 0.0001) and in a stepwise regression analysis on 15 variables possibly affecting cIMT, age (r^2^ = 0.3923, p < 0.0001), insulin clearance (r^2^ = 0.4564, p < 0.0001), systolic blood pressure (r^2^ = 0.4733, p < 0.0001), body mass index (BMI) (r^2^ = 0.4804, p = 0.002), gender (r^2^ = 0.4831, p = 0.013), and fasting insulin clearance (r^2^ = 0.4857, p = 0.030) turned out to be significant determinants of cIMT. In a cross-validated model resulting from this analysis, insulin clearance was found to be an independent determinant of cIMT (ß_st_ = − 0.16, p < 0.0001) even after adjusting for traditional CVD risk factors. Reduced insulin clearance may be an early marker of damage on the vasculature, independent of classical CVD risk factors. Reduced insulin clearance should be considered with regard to vascular insulin resistance.

## Introduction

Type 2 diabetes mellitus (T2D) promotes the development of atherosclerosis and, subsequently, increases the risk for cardiovascular disease (CVD)^1^. Enlarged intima-media thickness indicates structural changes in the vessel that contribute to atherosclerotic progression, whereby wall changes may be detectable several years prior to cardiovascular disease (CVD)^2,3^. One vessel bed that is easily accessible by ultrasound and therefore often used for measurement of the intima-media thickness are the carotid arteries (cIMT).

Underlying mechanisms that influence intima-media thickness are still under investigation. One prominent factor that associates with cIMT is elevated glucose: both fasting and postprandial glucose as well as long-term glycemic control correlates with risk for CVD^4^. This relation appears to be present not only in patients with overt diabetes but also in prediabetic state as well as in otherwise healthy individuals^5,6^. In addition, vascular damage appears to caused not solely by hyperglycemia, but also by insulin resistance and the associated hyperinsulinemia, which both are characteristics of T2D^7–9^. Insulin is released from the pancreas into the portal vein and approximately 50% is cleared during first pass through the liver, whereby C-peptide is not cleared by the liver. Reduced hepatic insulin clearance leads to elevated insulin concentrations in the systemic circulation^10^. Of note, reduced insulin clearance appears to be associated with atherosclerosis, independent of insulin-stimulated glucose disposal^11^. Though, the underlying mechanisms are still incompletely understood and the relative contribution of the different proposed pathomechanisms is yet unclear. Therefore, we systematically examined the relative contributions of glycemia, insulin secretion, sensitivity, and clearance as well as classical cardiovascular risk factors for early atherosclerosis, assessed by cIMT.

## Methods

### Participants and study design

Data from the Tübingen Lifestyle Intervention Program study were analyzed, which encompasses individuals at increased risk for T2D^12^. Participants with known diabetes mellitus prior to study inclusion or history of cardiovascular macrovascular disease, e.g., coronary heart disease or peripheral artery disease, were not included and all individuals underwent physical examination, laboratory testing as well as measurement of cIMT. Individuals were included in the study when they fulfilled at least one of the following criteria: a family history of type 2 diabetes mellitus, a body mass index (BMI) of greater than 27, and a previous diagnosis of impaired glucose tolerance or gestational diabetes. They were considered otherwise healthy according to results of a physical examination and routine laboratory tests. Data of 754 individuals were available, whereby 87 persons were excluded from the analyses due to incomplete oral glucose tolerance test (oGTT) (n = 11) data set or missing blood pressure (n = 76). Therefore, 667 individuals with a complete data set were available for analysis (see Table 1). From all participants, informed written consent was obtained and the local Ethics Committee of the Medical Faculty of the Eberhard Karls University of Tübingen, Germany, approved the study protocol.

**Table 1:** Characteristics of the study population.

	Total
Age (years)	44.1 ± 12.0
Gender (male/female)	250/417
NGT/IFG/IGT/IFG + IGT/diabetes	421/88/67/60/31
Body mass index (kg/m^2^)	30.6 ± 5.8
Waist circumference (cm)	98.2 ± 14.4
Systolic blood pressure (mmHg)	127.5 ± 17.7
Diastolic blood pressure (mmHg)	79.9 ± 11.4
Fasting blood glucose (mmol/l)	5.3 ± 0.6
2 h glucose-stimulated glucose (mmol/l)	7.0 ± 2.0
Fasting insulin (pmol/l)	55.7 ± 38.1
Fasting proinsulin (pmol/l)	5.5 ± 6.3
Fasting insulin clearance (pmol/l)	13.5 ± 5.2
Glucose-stimulated insulin clearance (AU)	6.7 ± 2.5
Insulin sensitivity, oGTT-derived (AU)	15.4 ± 9.5
Glycated hemoglobin (%/mmol/mol)	5.6 ± 0.5/37.5 ± 5.0
Total cholesterol (mmol/l)	5.1 ± 1.0
Triglycerides (mmol/l)	1.5 ± 1.0
LDL-cholesterol (mmol/l)	3.2 ± 0.9
HDL-cholesterol (mmol/l)	1.3 ± 0.3
hsCRP (mg/dl)	0.3 ± 0.3
Estimated glomerular filtration rate (ml/min/1.73 m^2^)	84.3 ± 18.9
cIMT (mm)	0.57 ± 0.13
Liver fat content (%)^a^	6.6 ± 6.9
Smoking (yes/no)^b^	92 / 509

Data shown as mean (± standard deviation).

^a^Liver fat content was available for 484 individuals.

^b^Smoking status was available for 651 individuals.

### Anthropometric characteristics, biochemical data, measurement of liver fat

Blood pressure was measured non-invasive according to Riva-Rocci after 10 min rest in a sitting position. After a 10 h overnight fast, venous blood samples were drawn at baseline and at time-points 30, 60, 90, and 120 min of a 75 g oGTT. The glucose-oxidase method was used to determine plasma glucose (Yellow Springs Instrument Co., Inc., Yellow Springs, Ohio). Fast venous blood samples were also used to determine high sensitive C-reacitve protein (hsCRP), triglycerides, total-, high density lipoprotein- (HDL) and low density lipoprotein- (LDL) cholesterol from plasma on the ADVIA XPT clinical chemical analyzer. Serum proinsulin concentrations were measured using an enzyme-linked immunosorbent assay (IBL, Hamburg, Germany) on a BEP 2000 analyzer. Plasma insulin and C-peptide were determined on the ADVIA Centaur XPT chemiluminescent immunoanalyzer (all instruments above from Siemens Healthineers, Eschborn, Germany). HbA1c measurements were performed using the Tosoh glycohemoglobin analyzer HLC-723 G8 (Tosoh Bioscience Tokyo Japan). Glomerular filtration rate was estimated using the Modification of Diet in Renal Disease formula as described previously and is given in ml/min/1.73 m^2^ of body-surface area^13^. Proton magnetic resonance spectroscopy (1H-MRS) of the liver was applied to measure liver fat content. MR imaging and 1H-MRS measurements were performed on a 1.5 T whole body imager (Magnetom Sonata, Siemens Healthcare, Erlangen, Germany) in the early morning after an overnight fasting. Liver fat content is given in % and data were available in a subgroup of 484 individuals.

### Assessment of insulin and glucose metabolism

The insulin sensitivity index was estimated as proposed by Matsuda and DeFronzo (ISI-Mats)^14^. Fasting insulin clearance was calculated as C-peptide_0_/Insulin_0_ and glucose-stimulated insulin clearance, referred to as insulin-clearance in this study, was calculated as AUC_C-Peptide(0–120)_/AUC_Insulin(0–120)_ during oGTT. Further fasting state- and oral glucose tolerance test (oGTT)-derived indices of insulin and glycemic metabolism were calculated as described before including oral disposition index^15,16^.

### Measurement of carotid intima-media thickness

The cIMT examiner was blinded to the study subject’s physical and laboratory findings. The examiner was experienced and gained knowledge in our vascular department by performing 4500 vascular ultrasounds per year with a wide range of vascular sites including cIMT. Measurement was performed in the early morning before starting oGTT with a linear ultrasound transducer (10–13 MHz; AU5 Harmonic, ESAOTE BIOMEDICA, Hallbergmoos, Germany). High-resolution ultrasound of left and right common carotid artery was performed in B-mode according to the European Mannheim carotid intima-media thickness consensus^17^. A mean of each side was calculated after performing three measurements of each side for reproducibility^18^. Considering known side differences, one mean was determined out of left and right mean cIMT and was used for further statistical analyses^19^.

### Statistical analysis

Normally distributed data are presented as means and standard deviations unless otherwise stated. Prior to analyses, we logarithmically transformed data that were not normally distributed to approximate normal distributions. To adjust for covariates and to identify independent associations, we performed multivariate linear regression analysis and effect sizes are reported as standardized beta coefficients (ß_st_). Stepwise analyses with fivefold cross validation were carried out, and results are given as k-fold r^2^. All numerical variables were used as a continuous variable. A p-value < 0.05 was considered statistically significant. The JMP 13.0 statistical software (SAS Institute, Cary, NC) was used for analyses.

## Results

**Figure 1:**
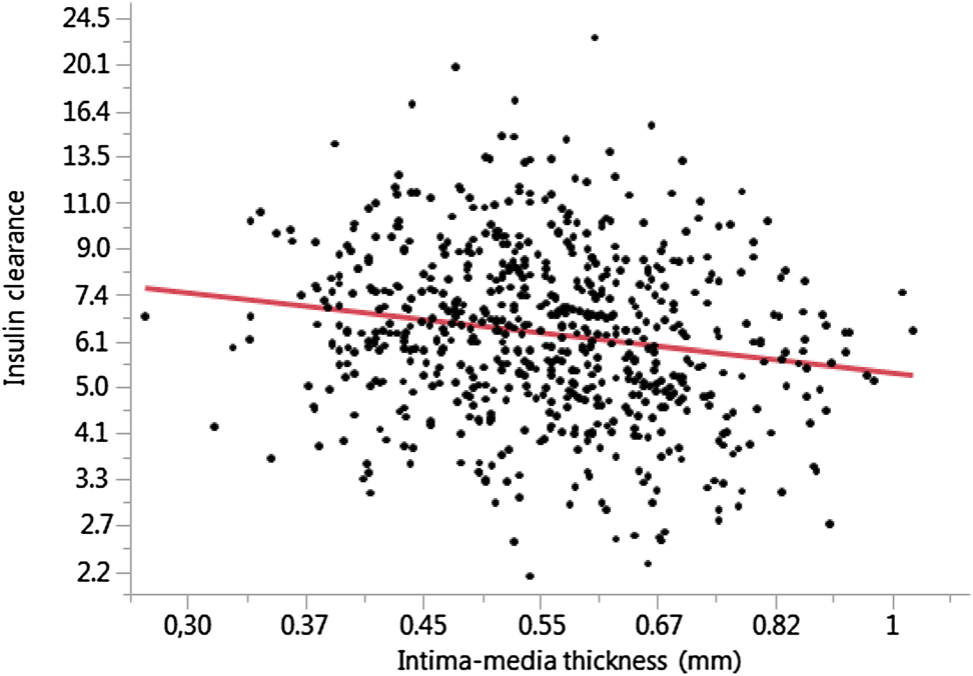
Univariate relationship between carotid intima-media thickness and glucose-stimulated insulin clearance.

We first addressed possible determinants of carotid intima-media thickness by applying univariate unadjusted analyses (see Table 2). In this approach, we confirmed classical factors associated with enlarged cIMT, such as increasing age, waist circumference, blood pressure, and dyslipidemia. Furthermore, glycemia and insulinemia were positively and insulin sensitivity negatively associated with cIMT (Table 2). While glucose-stimulated insulin clearance was inversely correlated with cIMT (Fig. 1), fasting state insulin clearance did not reach statistical significance (p = 0.05).

**Table 2:** Univariate associations of carotid intima-media thickness with demographic and metabolic characteristics.

Variable	ß_st_	*p* value
Age	0.63	** < 0.0001**
Body mass index	0.27	** < 0.0001**
Waist circumference	0.37	** < 0.0001**
Systolic blood pressure	0.37	** < 0.0001**
Diastolic blood pressure	0.28	** < 0.0001**
Fasting blood glucose	0.31	** < 0.0001**
Fasting insulin	0.20	** < 0.0001**
Fasting proinsulin	0.03	0.39
Fasting insulin clearance	− 0.08	0.05
Glucose-stimulated insulin clearance	− 0.17	** < 0.0001**
Insulin sensitivity, oGTT-derived	− 0.28	** < 0.0001**
Oral disposition index	− 0.22	** < 0.0001**
Glycated hemoglobin	0.27	** < 0.0001**
Total cholesterol	0.21	** < 0.0001**
Triglycerides	0.18	** < 0.0001**
LDL-cholesterol	0.18	** < 0.0001**
HDL-cholesterol	− 0.12	**0.003**
hsCRP	0.10	**0.013**
Estimated glomerular filtration rate	− 0.04	0.27
Liver fat	0.30	** < 0.0001**

A *p*-value < 0.05 was considered statistically significant and is given with bold values.

**Figure 2:**
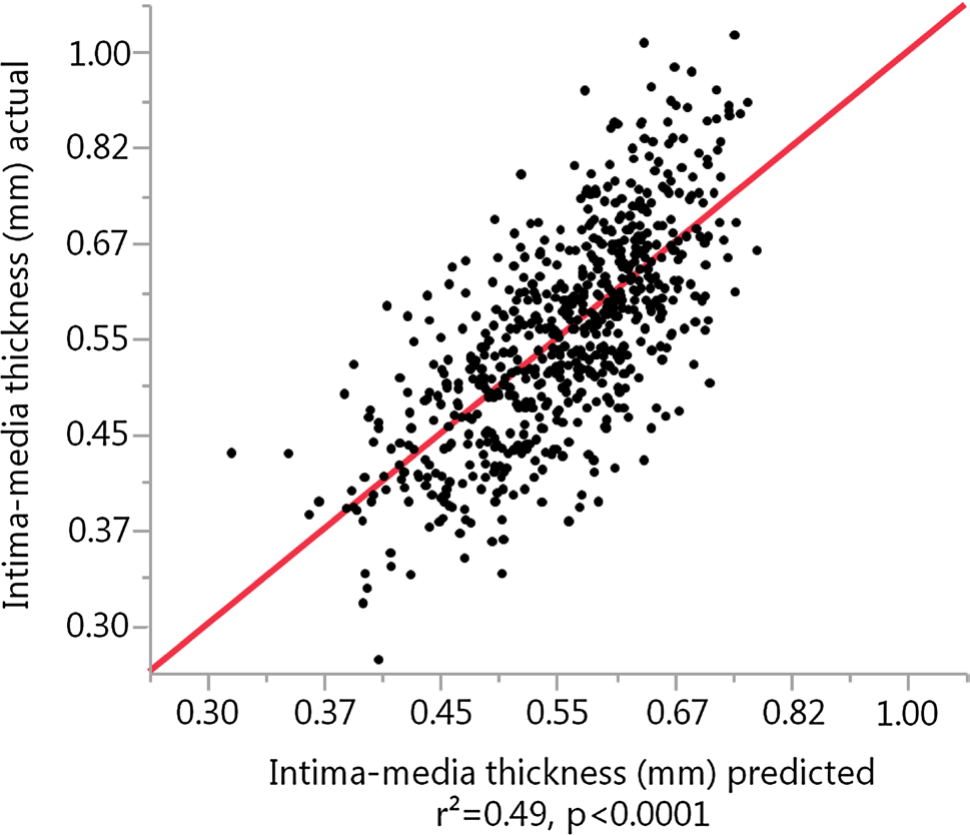
Multiple linear regression analysis of variables influencing intima-media thickness.

In order to identify the major determinants of cIMT, we performed stepwise multivariate regression analyses with cIMT set as the dependent variable and 15 independent variables (Table 3). Forward stepwise regression modelling with fivefold cross validation selected age, glucose-stimulated insulin clearance, systolic blood pressure, BMI, gender, and fasting insulin clearance as determinants of cIMT (Table 3). After age, glucose-stimulated insulin clearance was the strongest correlate of cIMT, even before traditional CVD risk factors such as systolic blood pressure and BMI. Based on this analysis, we constructed a statistical model for multiple linear regression analysis. In a model including age, both fasting as well as glucose-stimulated insulin clearance, systolic blood pressure, BMI, gender, glucose-stimulated insulin clearance was found to be related to cIMT as shown in Fig. 2 and Table 4, independent of the aforementioned variables. Age and insulin clearance were the traits most strongly and independently associated with cIMT (Table 5) even after additional adjusting for further cardiovascular risk factors and inflammatory parameters including LDL-cholesterol, smoking status, and hsCRP.

**Table 3:** Stepwise forward linear regression analysis with fivefold cross validation of variables possibly influencing carotid intima-media thickness with carotid intima-media thickness as the dependent variable.

Variable	k-fold r^2^	*p* value
Age	0.3936	** < 0.0001**
Glucose-stimulated insulin clearance	0.4588	** < 0.0001**
Systolic blood pressure	0.4768	** < 0.0001**
Body mass index	0.4832	**0.002**
Gender	0.4852	**0.013**
Fasting insulin clearance	0.4845	**0.030**
Glycated hemoglobin	0.4833	0.06
Proinsulin_120_/Insulin_120_	0.4840	0.23
hsCRP	0.4831	0.32
Proinsulin_0_/Insulin_0_	0.4833	0.42
AUC_Insulin(0–120)_	0.4836	0.37
Fasting blood glucose	0.4819	0.41
Insulinogenic index-2	0.4717	0.50
AUC_C-peptide(0–30)_/AUC_Glucose(0–30)_	0.4713	0.70
Insulin sensitivity, oGTT-derived	0.4710	0.49

A *p*-value < 0.05 was considered statistically significant and is given with bold values.

**Table 4:** Multiple linear regression analysis of variables influencing intima-media thickness.

Variable	Estimate (ß_st_)	Standard error	*p* value
Age	0.60	0.02	** < 0.0001**
Gender	− 0.08	0.01	**0.001**
Body mass index	0.11	0.04	**0.001**
Systolic blood pressure	0.12	0.05	**0.001**
Glucose-stimulated insulin clearance	− 0.16	0.02	** < 0.0001**

A *p*-value < 0.05 was considered statistically significant and is given with bold values.

**Table 5:** Multiple linear regression analysis of variables influencing intima-media thickness including further cardiovascular disease risk factors.

Variable	Estimate (ß_st_)	Standard error	*p* value
Age	0.62	0.02	** < 0.0001**
Gender	− 0.09	0.01	**0.003**
Body mass index	0.08	0.04	**0.017**
Systolic blood pressure	0.11	0.05	**0.001**
Glucose-stimulated insulin clearance	− 0.16	0.02	** < 0.0001**
LDL-cholesterol	− 0.01	0.02	0.71
hsCRP	0.06	0.01	0.06
Smoking	0.01	0.01	0.98

A *p*-value < 0.05 was considered statistically significant and is given with bold values.

We next performed subgroup analyses in subjects with newly diagnosed diabetes mellitus (n = 31), IFG (n = 88), IGT (n = 67), both IFG and IGT (n = 60) and with normal glucose regulation (n = 421). In univariate analyses, insulin clearance was significantly associated with cIMT in participants with newly diagnosed (ß_st_ = − 0.39; p = 0.03) and without diabetes mellitus (ß_st_ = − 0.11; p = 0.03) as well as IFG (ß_st_ = − 0.27; p = 0.01). In the subgroups with IGT and both IFG and IGT, this did not reach statistical significance (p = 0.16 and p = 0.23, respectively). In forward stepwise regression analyses, insulin clearance was an independent determinant of cIMT in subjects with newly diagnosed diabetes mellitus (r^2^ = 0.2828; p = 0.0378) and without diabetes mellitus (r^2^ = 0.4301; p < 0.0001). In persons with IFG, this was not significant. In the population excluding study subjects with newly diagnosed diabetes mellitus (n = 33), forward stepwise regression modelling revealed age (r^2^ = 0.3995; p < 0.0001) and insulin clearance (r^2^ = 0.4685; p < 0.0001) as the most strongly and independently determinants associated with cIMT.

Next, we were investigated gender and liver fat content as possible determinants influencing the above mentioned results in the entire cohort. No interaction between gender and insulin clearance on cIMT was detectable (p = 0.91). There was no interaction between liver fat and insulin clearance on cIMT, neither unadjusted (p = 0.72) nor after adjustment for age and gender (p = 0.24). Accordingly, insulin clearance was still associated with cIMT after adjusting for liver fat content, age, and gender (ß_st_ = − 0.25, p < 0.0001). Adding liver fat content in the above mentioned stepwise multivariate regression analyses including 15 variables with cIMT set as the dependent variable, liver fat content was not correlated with cIMT (r^2^ = 0.5147, p = 0.5307).

Furthermore, there was no association between estimated glomerular filtration rate and cIMT, neither in the entire cohort, nor after exclusion of participants with impaired kidney function (n = 48 with estimated glomerular filtration rate < 60 ml/min/1.73 m^2^; p = 0.27 and p = 0.8, respectively). Furthermore, there was no interaction between estimated glomerular filtration rate and insulin clearance on cIMT (p = 0.25).

## Discussion

In a cohort of well-characterized participants at increased risk for T2D or newly screening-diagnosed and yet untreated T2D, we examined the relative contributions of glycemic traits and classical cardiovascular risk factors for early atherosclerosis. We identified glucose-stimulated insulin clearance as a robust parameter that was associated with higher cIMT, independent of other investigated factors.

Similar to previous results, classical cardiovascular risk factors such as age and systolic blood pressure were also associated with higher cIMT in our cohort^20–22^. In contrast, glucose was not included among the most important covariates in the final model of our stepwise procedure. This was surprising, because elevated glucose is believed to be a major determinant of cardiovascular risk in patients with diabetes, and glucose appears to be also important in persons with normal glucose tolerance (NGT)^5,6,23^. Our results could implicate that the link between elevated glucose level and atherosclerosis could be secondary to other confounding factors that drive the association. We now identified an association between reduced insulin clearance and cIMT indicating a potentially link between insulin clearance and early vascular damage. While insulin clearance has been linked to cIMT in previous works, our findings now suggest that this association is independent of other major metabolic and cardiovascular risk factors like LDL-cholesterol or smoking^11^. When we performed subgroup analyses of the different glucose tolerance groups, insulin clearance was an independent determinant of cIMT in most subgroups. However, this did not reach statistical significance in some, presumably due to the very small sample sizes in these subgroups. As the associations were detectable in persons with normal glucose regulation as well as in those with newly diagnosed diabetes, the identified relations are most likely not depending on glucose metabolism.

The association of insulin clearance with cIMT could result from two localizations within the body: insulin clearance in the periphery and insulin clearance in the liver, whereby in our study liver fat content could not explain the independent association of insulin clearance with cIMT. In peripheral regions, reduced insulin clearance leads to higher circulating insulin concentrations^24^. At the vessel wall, insulin has pro- and anti-atherogenic properties. While signaling via the PI3K-Akt chain induces vasodilatation, signaling via the MAPK pathway promotes vasoconstriction, promotes proliferation of pericytes and smooth muscle cells, and attracts inflammatory cells. In case of hyperinsulinemia due to reduced insulin clearance, it is believed that there is a relative shift in signaling from the PI3K towards the MAPK pathway that ultimately results in endothelial dysfunction and vascular damage^8,25^. Anti-atherogenic factors are mediated by PI3K-Akt activation lead to activation of eNOS followed by the downregulation of adhesion molecules such as VCAM1. There is evidence that enhancing the aforementioned pathway improves endothelial dysfunction and decreases atherosclerosis even in the presence of hyperinsulinemia^26,27^. On the other hand, pro-atherogenic actions of insulin are mediated substantially by the Erk pathway, resulting in proliferation of the vascular smooth muscle cells. Changes in vascular tone may be a pathophysiological mechanism leading to a further reduction of insulin clearance. Reduced vascular permeability or blood flow results in an impaired transcapillary transport of insulin^28^. The link between insulin clearance and atherosclerosis is further underlined by findings from rodents: knockout of CEACAM1, the central protein for insulin clearance in the liver, causes hyperinsulinemia and marked endothelial dysfunction and formation of atherosclerotic plaques^29,30^. Though, we cannot exclude that a shared pathomechanism simultaneously induced atherosclerosis and reduced insulin clearance in the liver. As reduced insulin clearance preceds T2D, our current results indicate that besides elevated blood glucose level and the development of T2D, impaired insulin clearance can serve as an early marker for vascular damage^31,32^.

Our results substantially add to the recent observation of a link between insulin clearance and cardiovascular risk as we systematically examined its relative contribution independent of a large number of potential risk factors for subclinical atherosclerosis that had not been tested in this regard before^11^. A major strength of our study is the well characterized population at risk for diabetes but still without CVD. Though, our results must be confirmed in this patient group and should be followed up in prospective studies. A limitation of the study is that we assessed insulin clearance from an oGTT, that is not that strongly standardized as glucose clamp techniques. As drug intake could not be systematically analyzed, we can not exclude a possible influence of specific drugs on our results.

To sum up, reduced insulin clearance appears to be associated with sublinical vascular damage at a very early stage in individuals at risk for T2D. This is independent of insulin resistance and should therefore be considered as an additional factor that helps to identify persons at risk for cardiovascular disease.

## Abbreviations

AUC: Area under curve
BMI: Body mass index
CVD: Cardiovascular disease
cIMT: Carotid intima-media thickness
T2D: Type 2 diabetes mellitus
IFG: Impaired fasting glucose
IGT: Impaired glucose tolerance
HDL-cholesterol: High density lipoprotein-cholesterol
hsCRP: High sensitive C-reactive protein
HOMA-IR: Homeostasis Model Assessment Test of insulin resistance
LDL-cholesterol: Low density lipoprotein-cholesterol
NGT: Normal glucose tolerance
oGTT: Oral glucose tolerance test
